# Methodologies used to estimate tobacco-attributable mortality: a review

**DOI:** 10.1186/1471-2458-8-22

**Published:** 2008-01-22

**Authors:** Mónica Pérez-Ríos, Agustín Montes

**Affiliations:** 1 Department of Epidemiology, Directorate-General for Public Health, Galician Regional Health Authority, Santiago de Compostela, Spain; 2 Department of Preventive Medicine and Public Health, University of Santiago de Compostela, Santiago de Compostela, Spain; 3 CIBER Epidemiología y Salud Pública (CIBERESP), Spain

## Abstract

**Background:**

One of the most important measures for ascertaining the impact of tobacco on a population is the estimation of the mortality attributable to its use. To measure this, a number of indirect methods of quantification are available, yet there is no consensus as to which furnishes the best information. This study sought to provide a critical overview of the different methods of attribution of mortality due to tobacco consumption.

**Method:**

A search was made in the Medline database until March 2005 in order to obtain papers that addressed the methodology employed for attributing mortality to tobacco use.

**Results:**

Of the total of 7 methods obtained, the most widely used were the prevalence methods, followed by the approach proposed by Peto et al, with the remainder being used in a minority of studies.

**Conclusion:**

Different methodologies are used to estimate tobacco attributable mortality, but their methodological foundations are quite similar in all. Mainly, they are based on the calculation of proportional attributable fractions. All methods show limitations of one type or another, sometimes common to all methods and sometimes specific.

## Background

Since the association between tobacco and mortality was first discovered [1,2], the task of attributing a given number deaths to smoking has been and continues to be a controversial process, beset by limitations and questioned from different quarters, including the powerful tobacco industry. With the appearance of the successive revisions of the International Classification of Diseases (ICD), there has been considerable progress in the process of categorizing mortality, but little in methods for attributing mortality to risk factors such as tobacco. Obtaining reliable estimates of the impact of tobacco on mortality would facilitate to have a clearer picture of the problem caused by smoking and would be of help in the planning of health policy.

The task of quantifying smoking-attributable mortality has been performed mainly through indirect methods. This review sought to list and to describe the different methods of estimating mortality attributed to tobacco use, to indicate the principal methodological differences existing among them, and to identify the possible sources of variability in the results.

## Methods

In order to obtain papers that addressed the methodology employed for attributing mortality to tobacco use, a search was made in the Medline database until March 2005, using the terms, *mortality*, *attribut*,*method* and tobacco or smok*. The search was completed with a manual review of the bibliographic references cited by the papers retrieved and of other publications, such as the monographs published by the Centers for Disease Control and Prevention (CDC). The main inclusion criteria was the use of an epidemiological method to estimate attributable mortality. Papers describing mortality, such as cohort follow-up or mortality studies were excluded unless an epidemiological analysis had been used. Animal studies and communications presented at congresses were also excluded from the search.

The estimation of attributable mortality is also applied to other risk factors in addition to tobacco, such as alcohol consumption or obesity. In order to avoid the exclusion of valid methodologies the search was repeated without restricting it to tobacco or smoke.

## Results

The search yielded a total of 372 papers. Of these, 74 were finally included, as the rest did not apply mortality attribution methods. Some papers included more than one method. The unrestricted search, without the terms tobacco or smoke, did not furnish any new alternative methodology.

Revision of the 74 papers enabled us to identified 2 types of mortality attribution procedures for the specific case of tobacco. The first one is based on individual analysis of deaths to ascertain if tobacco use had any role in mortality. Only three studies applied this procedure [3-5]. The second is based on the application of indirect methods and constitutes the most commonly used methodology for attributing mortality. The total number of papers that employed this indirect methodology was 73, with 61 of these being yielded by the automatic and 12 by the manual search.

Seven indirect methods for estimating tobacco-attributable mortality were identified. The applied methodology in these 7 methods can be classified under four categories: Prevalence-based analysis (Prevalence-based analysis in cohort studies, prevalence-based analysis in case-control studies and the basic method), Peto and colleagues' method, methodologies based on the calculation of excess mortality (Garfinkel's and Roger's method) and predictive models (Prevent). The methods differ in terms of calculation processes, information requirement, data sources and assumptions required for their application. A summary of these methods is showed in Table 1. The main characteristics of the different indirect methods are described below.

**Table 1:** Methods used to estimate tobacco attributable mortality

Method	Data employed	Data source	Method applied to estimate mortality due to:	Weaknesses	Strengths	Estimations calculated
Prevalence-based analysis in cohort studyes/SAMMEC (n = 52)	Prevalence	National Statistics	Tobacco consumption, exposure to environmental tobacco smoke (ETS), obesity, alcohol intake,...	- Does not take latency into account.	- Worldwide use.	Attributable mortality for all causes.
	Relative Risks: Smokers, non-smokers and former smokers	Cohort study			- Application of risks other than CPS.	
Method proposed by Peto and colleagues (n = 6)	Relative Risks: Smokers and non-smokers	CPS II	Tobacco consumption.	- Assumes constant worldwide lung cancer mortality rates among never smokers.	- Worldwide use.	Attributable mortality for all causes.
	Lung cancer death rates: Global (non smokers + smokers + former smokers), non-smokers and smokers.	National Statistics/CPS II		- Assumes the same latency for all death causes related to tobacco.	- Mortality estimation in absence of smoking prevalence.	
				- Does not take into account former smokers.	- Takes latency into account for lung cancer.	
Basic method (n = 1)	Lung cancer death rates	National Statistics	Tobacco consumption.	- Partial view of attributable mortality (only used to estimate mortality by lung cancer).	- Takes into account induction time.	Lung cancer death rate attributable and not attributable to active smoking.
	Prevalence	National statistics/Estimated		- Use of constants.	- Estimates smoking-adjusted RR in different time periods.	
	Lung cancer relative risk	Calculated		- High need of information.		
	Packs of cigarettes smoked	National statistics/Estimated		- Rate ratios for former smokers.		
	Age of starting/giving up tobacco consumption	National statistics/Estimated		- Assumes constant worldwide lung cancer mortality rates among never smokers.		
	Constants	Previous studies				
Prevent method (n = 2)	Composition of the population	National Statistics	Tobacco consumption and general scenarios of effective health promotion.	- High need of information.	-Takes into account the multiplicity of cause or effect.	Attributable mortality for all causes.
	Mortality (population) and birth (women) rates	National Statistics			- Proportional decrease in risk reduction related to time.	
	Latency and delay	Previous studies			- To measure the results of intervention policies.	
	Time-Tendency of tobacco consumption.	Personal interviews				
	Relative risks	CPS II				
Prevalence-based analysis in case-control studyes (n = 4)	Mortality observed	National Statistics	Tobacco consumption and exposure to ETS.	- Case-control study design.	- Specific risk dates.	Attributable mortality for all causes.
	Exposure prevalence: case or controls	Case-control study		- Recall bias.		
	Odds Ratios	Case-control study				
Garfinkel's method (n = 2)	Mortality observed	National Statistics	Tobacco consumption and alcohol intake.	- Partial view of the attributable mortality (only used to estimate cancer mortality).	- Necessary dates are few.	Cancer deaths attributable to smoking.
	Cancer mortality rates in non smokers.	American Cancer Society		- Assumes constant worldwide cancer mortality rates among never smokers.	- Does not use risks or prevalence.	
Rogers' method (n = 1)	Mortality observed (all causes)	National Statistics	Tobacco consumption.	- Availability of mortality registries.	- Risks calculated ad hoc.	Attributable mortality for all causes.
	Prevalence (7 categories)	Surveys		- Has a population representative survey about health-risks.	- The population division is more reliable.	
	Odds Ratios	Discrete-time hazard models		- Assumption: smoking status remains steady since the survey about health-risks.		

### a) Prevalence-based analysis

Prevalence-based analysis or prevalence-risks models are based on the different distributions of the risk of dying from various tobacco-related diseases in relation to the prevalence of tobacco consumption in the population.

To apply these methods it is necessary to know the prevalence of smoking in the study population, the total number of deaths due to diseases causally related to tobacco use, and a measure that summarizes the increased risk of dying due to these causes among smokers and ex-smokers.

We can distinguish 3 methods due mainly to data source:

#### - Prevalence-based analysis in cohort studies

This method is the most widely employed in the literature [4,6-51].

Attributable deaths are calculated for each cause of mortality using the following formula:

AM = OM * PAF;

where AM is the mortality attributed to tobacco, OM the observed mortality, and PAF the population attributable fraction.

To calculate PAF, different methods exist [52,53], though the most widely used is based on the formula proposed by Levin [54] which divides the population into various categories according to tobacco use (non-smokers, ex-smokers and smokers):

PAF = ((p_0 _+ p_1_RR_1 _+ p_2_RR_2_)-1)/(p_0 _+ p_1_RR_1 _+ p_2_RR_2_);

where *p*_0_, *p*_1 _and *p*_2 _represent the prevalence of non-smokers, smokers and ex-smokers, respectively. RR_1 _and RR_2 _refer to the risk of dying for any cause of smokers and ex-smokers respectively compared to a baseline population of non-smokers.

Data are drawn from registries in the case of observed mortality and from surveys in the case of smoking prevalence. The relative risks (RRs) employed in the calculations are extracted mainly from the prospective cohort study conducted by the American Cancer Society, i.e., the Cancer Prevention Study II (CPS II) with follow-up at 4 [55] and 6 [56] years.

A modification of this method was proposed in the 1992 Surgeon General's report "Smoking and Health in the Americas" [57]. The authors created an index for measuring the smoking maturity in a population, based on a comparison of lung cancer rates. This index is multiplied by the disease-specific PAF to obtain an adjusted disease-specific PAF for a country.

The CDC's SAMMEC (Smoking-Attributable Mortality, Morbidity, and Economic Cost) computer software application [58] uses this methodology. SAMMEC is a software package commonly used in the United States to estimate attributable mortality due to smoking, years of potential life lost and indirect mortality costs. SAMMEC computes PAF automatically after the user includes prevalence of tobacco consumption. Furthermore, the user must supply the number of deaths by 5-years age groups from 35 or older, for each smoking-related diagnosis. Estimations from SAMMEC can include attributed deaths to fires and secondhand smoke. The Simsmoke model, a model that predicts the effect of policies on smoking rates and deaths attributable to smoking, uses this computer application to estimate deaths attributable to smoking.

Apart from being employed for calculating mortality due to tobacco use, this method has also been used for estimating mortality associated with exposure to environmental tobacco smoke [59-61], alcohol intake [8,18,21,24,29,62-65], illicit drugs [18,21], obesity [66,67], oral contraceptive use [68], hypertension status [69], cardiovascular processes [70], and diabetes status [71].

#### Prevalence-based analysis in case-control studies

Employing a similar calculation procedure to the previous method, this one emerged as a consequence of the objections raised by certain researchers about using RRs to estimate smoking attributable mortality from other countries [72]. This method has been used to estimate mortality attributable to tobacco use [73-75] in China when the epidemic was still in the initial phase.

To apply this method, it is necessary to know the total deaths for all causes among subjects aged 35 years or more for a given period of time. By interviewing survivors, information is collected retrospectively on smoking habits of deceased subjects 15 years before their death. Based on a case-control study risks are estimated.

Once these risks obtained, the population attributable fraction (PAF) can then be calculated, applying the formula:

PAF = P*(1-(1/RR));

where P is the proportion of deaths occurring among smokers and RR the relative risk calculated as OR after completion of a case-control study.

When the PAF has been calculated, deaths attributed to tobacco use (AM) in the study population can be estimated as follows:

*AM *= OM*PAF

#### Basic model

The Basic model [76] was originally applied in the setting of occupational cohort studies, to assess confounding generated by tobacco use.

This model has been employed in only one study [76] to estimate non-tobacco-attributable lung cancer mortality rates. Unlike the previous methods, different processes are specified here for calculating the RRs of lung cancer in smokers and ex-smokers versus non-smokers. From a paper previously published [77] authors adapted two functions to compute rate ratios. Both of them take into account duration and intensity of smoking.

Lung cancer rate not attributable to smoking (I_o_) can be calculated as follows:

$I_{o}=\frac{I}{P_{0}+P_{1}RR_{1}+P_{2}RR_{2}}$; where I is the overall lung cancer mortality rate.

### b) Method proposed by Peto et al

Although this method could be defined as a prevalence-risk model, particularities in its calculation procedure and assumptions would classify it separately.

Peto et al. [78,79] established a method for estimating tobacco-related mortality in which the need for data, especially for lung cancer estimates, is less demanding than in any of the other procedures reviewed. These authors postulate that lung cancer mortality is an indicator of the maturity of the smoking epidemic in a population, and thus, that tobacco-attributable mortality can be estimated by lung cancer mortality. This model may estimate mortality independently of the prevalence of smoking in the study population.

To apply this method, one needs to know the age- and sex-specific lung cancer mortality rates in the target country (C_LC_) and also in never-smokers of the same population (N_LC_), the relative risks for all diseases and disorders causally related to tobacco, except lung cancer; and the cause-specific lung cancer mortality rates in smokers (S*_LC_) and never-smokers (N*_LC_), taken from a cohort study. Peto et al used data drawn from the CPS II.

The calculation of the estimated tobacco-attributable mortality has two well-defined procedures: one to estimate attributed lung cancer mortality, and the other to estimate mortality attributable to all the remaining diseases with an established causal relationship [55,56].

The sex- and age-specific proportions of lung cancer deaths attributable to tobacco are obtained through the following formula:

(C_LC _- N*_LC_)/C_LC_

For the remainder of the diseases causally associated with tobacco use, the calculation process is different. The first step is to estimate thesummarized smoking prevalence or smoking impact ratio (SIR), which summarizes the history of tobacco use in the population by age and sex. SIR was defined as population lung-cancer mortality in excess of never-smokers, relative to excess lung-cancer mortality for a known reference group of smokers, adjusted for differences in never-smoker lung-cancer mortality rates across populations [80]. Smokers in the study population are converted into equivalent of smokers in the reference population. The formula used for its calculation is:

$$SIR=\frac{C_{LC}-N_{LC}}{S_{LC}^{*}-N_{LC}^{*}}$$

This formula is used in all populations where lung cancer mortality rates among non-smokers are unknown. Where these data are available one needs to normalize the formula [80].

The second step of this process consists of computing the population etiological fraction (PEF) on the basis of the previously calculated summarized prevalence (SIR) and the relative risks of dying due to the respective causes (RR), by age group and sex, as per the CPS II.

PEF = SIR(RR - 1)/(1 + (SIR(RR - 1)).

To ensure that the resulting PEF was not exaggerated by excessively high RRs, Peto et al. adjusted the formula proposed by Levin [54] by replacing the 1 in the denominator by a 2.

Once the RRs from the CPS II had been re-analyzed and their robustness confirmed, the earlier reduction was viewed as excessive, and a reduction of 30% applied instead [81]. In countries like China, where country-specific risks are available, the reduction applied is lower.

The last step in this procedure would involve applying the following formula: *AM *= OM*PEF, in order to obtain the estimation of attributed mortality, AM, in accordance with the PEF previously calculated and the observed mortality, OM.

This method has only been applied to estimation of tobacco-attributable mortality [11,78,79,81-83].

### c) Excess mortality methods

#### Garfinkel's method

Cancer deaths due to smoking are calculated as the difference between observed and expected deaths in a population. To apply this method, age- and sex-specific cancer mortality rates are needed, and age- and sex-specific cancer mortality rates for non-smokers are computed on the basis of the CPS study [84]. The expected deaths are related to the number of deaths that would occur if the whole population was formed by non smokers. To calculate the expected number of deaths, the follow-up over 12 years of the never smokers enrolled at the CPS I study was employed and death rates for cancer were computed. These rates were applied to the estimated number of person-years of exposure for non-smokers to obtain the expected number of deaths for each cancer. The attributable fractions calculated in this way were similar to those yielded by the CPS [85]. Garfinkel's method was applied to estimate cancer mortality attributable to tobacco use [85-87].

#### Rogers' method

The method proposed by Rogers et al. [88] combines prevalence and mortality risk rates in order to offer more precise estimates of smoking attributable mortality. This calculation procedure attempts to avoid some problems related to previous methods as 1) the use of risks derived from selected populations, 2) the absence of adjustment for confounding factors or 3) the classification of the smoking status in crude categories without attending to the number of cigarettes smoked by former and current smokers. At first, age-specific smoking prevalence and mortality risks were estimated. The authors define 7 population groups distinguished by reference to the amount of cigarettes smoked (*p) *and classifies them by sex and age-group: non-smokers, light smokers, moderate and heavy smokers, light ex-smokers, and moderate and heavy ex-smokers. To determine the risk of death due to cigarette smoking, Roger et al. matched data of a health survey to mortality data. Discrete time hazard models were employed to compute the risks.

The next step is to determine how many people exist in each smoking status (n):

*n *= p*Pop, being Pop the age-specific population in the area studied.

The last step is to estimate the excess risk of death (R) of each smoking status relative to never smokers:

m_x,c _- m_x,n_, where m_x,c _is the age-specific central death rate for each smoking status and m_x,n _is the age-specific central death rate relative to never smokers.

Finally the excess number of deaths is calculated as follows:

ED = ∑n*(m_x,c _- m_x,n_), in the different ages-groups considered.

This method has been used once to estimate tobacco attributable mortality [88].

### d) Predictive models

These models are represented essentially by one model: the Prevent model [83].

The Prevent simulation model [83] was developed in 1988 in The Netherlands and is regarded as being a multifactorial generalization of the etiologic fraction. It has been used basically to predict mortality due to various causes, including tobacco [89]. The methodology used allows, among other factors, for a temporal dimension to be considered and takes into account the possibility of a risk factor to associate with more than one disease and a disease to associate with more than one risk factor. The process of calculation is tedious and needs knowledge of multiple data, such as birth- and mortality-rate series or the likelihood of dying at different ages for each sex [90]. The calculation procedure was described in detail in a phD dissertation [90] and is summarized elsewhere [83,91]. Due its scarce use, the calculation procedure is not described in this paper. However it is important to introduce two epidemiological effect measures that this method uses: the "potential impact fraction" and the "trend impact fraction". Both are indicators of the reduction in the incidence of a disease in the population studied, the former reflects changes in the evolution of a disease after an intervention and the latter is referred to autonomous or natural trends.

## Discussion

This paper constitutes, to our knowledge the first methodological review of procedures for estimating smoking-attributable mortality. In the context of decision-making it is essential to know, albeit approximately, the impact that a given risk factor has on the mortality of a population. Estimation of tobacco-related mortality is not confined to one procedure alone, inasmuch as any of the different methods outlined above can be used for the purpose.

Despite the fact that different methodologies have been found, the foundations of more of them are the same and only few differences arise in the calculation procedures (Table 2). Data availability has been taken into account when choosing a method and also methodology limitations and assumptions have to be considered. Some of them are described below.

**Table 2:** Methodologies' modifications taking into account prevalence-based analysis in cohort studies as base method

	**Peto's et al method**	**Prevalence-based analysis in case-control studies**	**Basic method**
**Variation respect prevalence-based analysis in cohort studies**	Problem: Smoking prevalence is a poor proxy for cumulative hazards of smoking. Solution: Defining SIR (Smoking impact ratio or Synthetic prevalence) authors avoid prevalence limitations.	Problem: RR extrapolation to different populations than the original is inconsistent. Solution: Designing a case-control study OR could be assessed.	Problem: RR extrapolation to different populations than the original is inconsistent. Solution: RR can be estimated applying a calculation procedure.
**Calculation procedure**	$SIR=\frac{C_{LC}-N_{LC}}{S_{LC}^{*}-N_{LC}^{*}}$ where C_LC_, N_LC_, S*_LC_, N*_LC _are age-sex specific lung cancer mortality rates for smokers and never smokers in the study and in the reference population (*).	$OR=\frac{a_{1}b_{0}}{a_{0}b_{1}}RR=\frac{a_{1}(1-p_{1})}{a_{0}p_{1}}$ where p_1 _is the prevalence between the cases a_1 _is exposed cases b_1 _is exposed controls a_0 _is non exposed cases b_0 _is non exposed controls	Packs-function in smokers RRs = 1 + ac((t - 5))-t_0_) Multistage-function in smokers RRs = 1 + [(t - 5)^4.5 ^+ ac(1 + 2ac)((t - 5)-t_0_)^4.5 ^+ 2ac((t - 5)^4.5^-t_0_^4.5^)]/(t - 5)^4.5^ Where *a *is a constant, *c *is the number of packs of cigarettes smoked per year, *t *is the current age and *t*_0 _is age at start of smoking. In former smokers t_1 _replaces (t - 5) and t_1 _is age at stop smoking.

The first limitation affecting intercomparison of methods and studies stems from the absence of a universal definition of the categorization of tobacco use. The publications analyzed furnish different definitions of "smoker", "non-smoker" and "ex-smoker" [6,92-95], something that inevitably determines the result of the estimation [96].

To view smokers as a single entity could lead to a distorted mortality estimate, since failure to take account of the number of cigarettes smoked, age at initiation, years of smoking and other variables that could modify risks values can occur. It would thus be interesting to explore tobacco use in the studied populations [88]. A correct classification of ex-smokers is very important for estimating and predicting mortality attributable to tobacco use. To avoid overestimation of attributed mortality, Anthonisen [97] proposed that account must be taken of the decrease in risk that takes place at 15 years after quitting the habit. But this decrease is also determined by the subject's age at cessation [98,99], the duration of smoking [100] and the cause studied. The fact that this information was not expressly gathered in the majority of surveys means that mortality among ex-smokers may be overestimated. This problem is solved, at least in part, by ex-smokers reclassifying themselves as non-smokers after the elapse of a long time without smoking [101].

The second limitation, present mainly in the proportional method, resides in their reliance on current smoking prevalences to reflect mortality occasioned by tobacco use in previous years. Knowing current smoking prevalence could be a great help when it comes to predicting future mortality, but not present[102]: indeed, knowing the prevalence of tobacco use in any given year could help predict lung cancer mortality in 20 years' time [103]. As yet, this problem has no easy solution, due to the absence of historical series of smoking prevalence in most countries. Moreover, even if such series were to exist, lack of knowledge of the latency and induction times for each of the tobacco-related causes of death would constitute another problem. The use of current prevalence may overestimate or underestimate the attributable mortality. In countries where the prevalence is decreasing, as U.S.A. or some European nations, the use of current prevalence is conservative in the proportional attribution method. The opposite occurs in countries where prevalence is increasing. Given the unavailability or inaccuracy of prevalence data, and emphasizing that current prevalence is a poor proxy for cumulative hazards of smoking, the knowledge of the period of time from tobacco consumption until mortality related to this use it is necessary.

Ascertaining the induction period might be feasible if only one specific component cause was active in triggering the disease. However, if one allows for the presence of more than one component cause, then each may have its own induction time; furthermore, the action of effect modifiers could alter the induction period [104]. It would therefore seem that ascertainment of the induction period is complicated; nevertheless, ascertaining the latency period is no easy matter either, since it varies according to the diagnostic methods. What should be clear, however, is that an induction time is needed for tobacco to cause harm, and it is for this reason that the age ranges between 30–35 years are considered the time to begin measuring the effects of exposure. Measuring such effects without taking into account an induction time could lead to overestimated mortality results. On the other hand, some authors [88] feel that ignoring mortality under the age of 35 years may give rise to underestimates of mortality figures, due to the existence of individuals who started smoking at early ages.

Peto et al. avoided the problem entailed in prevalence-dependent methods of attribution. For the application of their estimation procedure, lack of knowledge of the tobacco consumption or latency and induction periods are no a limitation. But this method has not been exempt from criticism [25,105-110] directed, mainly, at the calculation of summarized prevalence. Some of these critics were supported by the tobacco industry, which tried to undermine the studies focused on estimations of mortality attributable to tobacco consumption. Peto and colleagues defined synthetic prevalence as an indicator that summarizes a population's smoking history, and calculate it by assuming CPS II data on lung cancer mortality rates among smokers and non-smokers to be valid. The use of these 2 sets of data gave rise to numerous criticisms that highlighted the low population representativeness of the CPS II [25,107,111,112]. Most of the population included in this cohort study was middle class, which may result in lung cancer mortality in non-smokers being underestimated [88] leading, in turn, to an overestimation of lung cancer mortality attributable to tobacco use and, by extension, to an overestimation of the summarized prevalence [25]. To justify their validity and universality, these data were compared with those yielded by the study that targeted British physicians [93]; despite the fact that the results obtained were similar, no conclusion could be drawn, since the representativeness of this latter study was also limited. The only thing that could be said was that the lung cancer mortality rate among non-smokers had not varied over the years[111]. Nonetheless, in countries where the use of coal is widespread, lung cancer mortality among non-smokers is higher, and thus the data, rather than being drawn from the CPS II, have been drawn from a local study [72].

The third limitation centers on the absence of world-wide risk indicators that would reflect the degree of association between tobacco and smoking related-causes of mortality. The most widely used effect measure is RR, and a sensitivity analysis has shown that changes in its value lead to a greater impact on the estimation of mortality than do changes in prevalence [102]. Although drawn from different sources, the RRs used in the various studies mainly came from the CPS II [55,56]. Applying these risks to populations other than that of the USA aroused criticism because, *inter alia*, of their only being adjusted for age and sex, and because of the difficulty inherent in assimilating identical tobacco consumption and genetic variability patterns, or the same influence of confounding factors or effect modifiers. A solution to these problems was sought through a re-analysis of the data [9,113-115], and the RRs were shown robust. Notwithstanding this, the criticisms continued unabated [116].

The risks obtained from the CPS II are plausible in the light of current knowledge [25] and have been extrapolated [117] to different EU countries, in absence of other high quality indicators. Nevertheless, other authors have chosen to apply RRs which are drawn from studies with less robust designs or possibly inconsistent with present knowledge.

A fourth limitation of the attribution methodology is the uncertainty present in the relationship between exposure, tobacco use, and different causes of death. While lung cancer was the first disease to be causally associated with tobacco use, many studies have observed more causal associations. The latest report of the Surgeon General [56] has added 2 further causes of mortality that had not been considered to date, i.e., stomach cancer and acute myeloid leukemia and excludes hypertension.

Some methods have been compared by applying them in the same population. Published comparisons are the individual analysis and SAMMEC [3,4], Peto and Prevent methods [83], Peto and proportional attribution method [11], and Garfinkel's and proportional attribution method [85]. The results obtained in all of these comparisons have proved to be similar estimations, thereby conferring validity on the respective methodologies. Observational epidemiology and, despite their limitations, the use of the above-described calculation procedures offer a good approximation of the impact of tobacco on the mortality of a population [4].

## Conclusion

Prior to conducting a study on estimation of tobacco-attributable mortality, it is essential to assess which method is best suited to the type and quality of the available information.

When the mortality estimation objective is going to be the knowledge of tobacco impact on a population, it is important to take into account all the diseases related with consumption. For this reason, the applications of methodologies that involve all the causes of disease are important. These methodologies are: Prevalence-based analysis in cohorts and in case-control studies, Peto et al. and Roger's methodology. All of them supply accurate and reliable estimations of mortality attributed to tobacco consumption.

The absence of a simulation study involving and comparing all calculations procedures do not allow us to recommend a method over other one.

These types of methods furnish estimates that constitute valuable information and help forming a more accurate picture of the problem that smoking poses to world health.

## Competing interests

The author(s) declare that they have no competing interests.

## Authors' contributions

MPR has made substantial contributions to conception and design, acquisition and reading of papers, and also have been involved in writing the manuscript AM has made substantial contributions to conception and design and to critically review the manuscript for important intellectual content; and have given final approval of the version to be published.

## Pre-publication history

The pre-publication history for this paper can be accessed here:


